# Healthcare equity in focus: bridging gaps through a spatial analysis of healthcare facilities in Irbid, Jordan

**DOI:** 10.1186/s12939-024-02120-8

**Published:** 2024-03-12

**Authors:** Bushra Obeidat, Sally Alourd

**Affiliations:** Department of Architecture, College of Architecture and Design, Jordan University of Science and Technology, 3030, Irbid, 22110 Jordan

**Keywords:** Healthcare distribution, Healthcare services, Jordan, Accessibility, Spatial analysis, Geographic information system (GIS)

## Abstract

In the Irbid Governorate, Jordan, equitable healthcare facility distribution is vital to ensuring healthcare accessibility and improving public health outcomes. This study investigated the spatial distribution, accessibility, and conformity of healthcare facilities to the Ministry of Health standards to identify areas requiring improvement. Using geographic information systems (GIS), three spatial analyses were conducted: nearest neighbor analysis, buffer analysis, and service area analysis. These analyses comprehensively assessed the healthcare landscape, revealing a random spatial distribution pattern of healthcare facilities; and indicating an absence of structured organization. The buffer analysis revealed concentrations in specific regions, while others were underserved. The Service Area Analysis revealed significant healthcare access challenges, especially in remote areas. The healthcare resource distribution of the Irbid governorate fell short of national and international standards, emphasizing the need for improvements. To address these disparities, policymakers and healthcare authorities should focus on equitably redistributing resources, tailoring allocation to local needs, improving remote area infrastructure, and refining government policies. Continuous monitoring and evaluation are imperative to ensure alignment with international standards and achieve healthcare equity. The insights from this case study provide valuable guidance for regions facing similar healthcare distribution challenges.

## Introduction

In the realm of global healthcare, the provision of adequate healthcare facilities has long been a paramount concern for human societies [7, 31]. Indeed, the health and well-being of individuals are central to the progress and prosperity of any society, making healthcare services an essential pillar of societal growth [8, 28]. Strategic planning and harmonization of these services with urban development are critical in addressing the evolving healthcare needs of growing populations [7, 36].

Enhancing the level of public health necessitates a geographical reconfiguration of healthcare facilities. This approach entails not only ensuring that these facilities are as accessible as possible in densely populated areas but also necessitating the establishment of new, carefully selected healthcare sites tailored to the specific healthcare demands of the populace [16]. Nevertheless, the equitable distribution of healthcare resources remains a significant challenge in numerous nations. Studies jointly conducted by the World Health Organization (WHO) and the World Bank (WB) reveal a disconcerting reality, with at least half of the world’s population lacking access to even basic healthcare services [35].

In response to this urgent issue, countries across the globe have developed criteria for the allocation of healthcare resources, placing a strong emphasis on the comprehensive and equitable distribution of healthcare facilities to foster social justice and ensure equal access [27]. In the Jordanian context, the Ministry of Health (MoH) is tasked with establishing standards for healthcare facility distribution [34]. In various governorates of Jordan, challenges are evident, including the scarcity of services, the emergence of informal settlements, escalating traffic congestion, and underserved residential neighborhoods [26].

Considering these pressing issues, concerted scholarly efforts have emerged to address the multifaceted challenges inherent in healthcare facility distribution and urban expansion. Achieving the optimal balance necessitates the alignment of perspectives between administrative and planning authorities on the one hand and the expectations of urban residents who require healthcare services on the other hand. Such efforts are further constrained by the need to adhere to established guidelines and scientific principles concerning land use, population dispersion, distances between healthcare facilities, and transportation accessibility [21].

Against this backdrop, this study seeks to illuminate the spatial distribution of healthcare facilities within the Irbid Governorate of Jordan. Its primary objectives include the analysis of existing healthcare services within the governorate and the assessment of their conformity with spatial planning standards delineated by the Jordanian government. Furthermore, spatial analysis will be harnessed to construct a comprehensive database that captures these healthcare services. This research endeavors to identify existing challenges and gaps in the present distribution of healthcare facilities and subsequently aims to provide recommendations for their resolution.

The analysis leverages geographic information systems (GIS) software and spatial statistical techniques incorporated into the GIS Arc software, facilitating an exhaustive examination of health center distribution patterns and spatial relationships. This research endeavors to contribute to the broader discourse on healthcare resource allocation and urban development, shedding light on a critical issue in Jordan and offering insights into the relevance of healthcare planning and spatial strategies in regions grappling with similar challenges.

### Healthcare landscape in Jordan

Within the healthcare landscape of Jordan, a multifaceted tapestry of service providers takes center stage, encompassing the public, private, international, and charitable sectors. The public sector, a cornerstone of the nation's healthcare apparatus, comprises the Ministry of Health, the Royal Medical Services, university hospitals, and the Center for Diabetes, Endocrinology, and Genetics [21]. Coexisting with the public sphere, the private sector boasts a network of hospitals, clinics, and numerous private healthcare practitioners. The international and charitable sectors add their unique contribution to the mosaic of healthcare services in the country, disseminating aid through clinics established by the United Nations Relief and Works Agency for Palestine Refugees in the Near East (UNRWA), the United Nations High Commissioner for Refugees (UNHCR), and various charity associations [21].

**Fig. 1:**
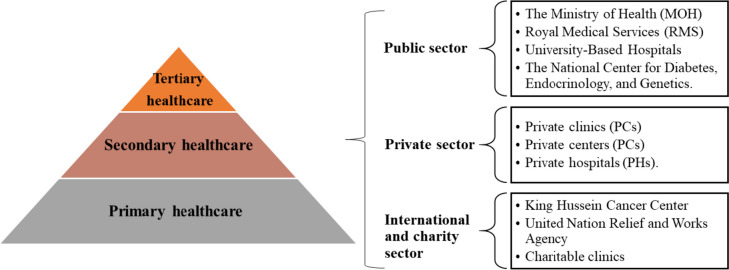
Level of care in Jordan and health sector components. Source: Ministry of Health

The Jordanian government's healthcare policy involves its mooring a system categorized into three hierarchical service levels, encompassing primary healthcare and tertiary care (Fig. 1) [21]. At the foundation of this structure lie primary healthcare services, built upon the principles of comprehensive healthcare delivery. This tier encompasses fundamental preventative and curative healthcare measures, such as health education, reproductive healthcare services, water and food safety initiatives, environmental health programs, early detection of chronic, genetic, and congenital diseases, mental health support, and services tailored to individuals with disabilities. The gamut of primary care also extends to domains such as school health, occupational health, the control of communicable diseases, dental health, and specialized healthcare services for individuals with distinct needs [21].

Crucially, these primary healthcare services are orchestrated through a network of health centers, maternity and childhood centers, and dental clinics administered by the Ministry of Health. The private sector also plays a pivotal role in augmenting this essential foundation of care, contributing via its array of clinics. Moreover, international agencies such as the UNRWA have further extended the reach of primary healthcare services to communities in need.

Delineated above primary healthcare, the landscape unfolds into the realms of secondary and tertiary healthcare facilities, characterized by a commitment to delivering highly specialized and efficient services conforming to global standards. Jordan's secondary and tertiary healthcare services are upheld by a constellation of public and private-sector hospitals dispersed across the kingdom's governorates [21]. These are exemplified by the intricate network of university-based hospitals, key players in the country's healthcare sector.

The Ministry of Health assumes the mantle of delivering secondary and tertiary healthcare services, leveraging government hospitals spanning the length and breadth of the kingdom. This extensive reach even extends to remote areas through the Royal Medical Hospitals. Educational institutions likewise partake in this venture, with Jordan University Hospital and King Abdullah University Hospital serving as essential contributors to the provision of secondary and tertiary curative services. Notably, the private sector's involvement is also integral to this tier of healthcare delivery [21].

This intricate web of healthcare providers forms the foundation for the healthcare system in Jordan, operating across various levels and sectors to address the diverse healthcare needs of the population.

### Healthcare facility planning and regulation in Jordan

The allocation and geographical distribution of healthcare facilities are pivotal for ensuring the accessibility and coverage of healthcare services [1, 17, 24, 32]. It is a complex task in which the population size and the radius of service distances constitute the center stage. Notably, it is feasible to anticipate the requisite number of healthcare facilities based on local population demographics [17, 24]. Each country crafts its own unique standards and guidelines for healthcare facility distribution, attuned to the nuances of its healthcare system [27].

Healthcare facility planning in Jordan is a multifaceted undertaking, featuring tailored planning regulations for two primary categories: health centers and hospitals [21].

#### Health centers

Health centers serve as the epicenter of medical services, delivering primary healthcare services at the neighborhood and village levels. The mission of these centers is to provide healthcare services for families within their scope, oversee the health status of each family member, offer curative and preventive services, and facilitate referrals to specialized medical centers when required [21]. This category encompasses comprehensive, primary, and sub-health centers (Table 1).

A. Comprehensive health centers provide a wide range of primary healthcare services, including general medicine, primary health care, maternity and childhood services, dental care, laboratory services, X-rays, specialized treatment, and emergency care. According to the Jordanian Ministry of Health, comprehensive health centers are established in areas with populations exceeding 5,000. Moreover, the distance from residents is regulated, with a minimum service radius of 2 kilometers and a maximum of 5 kilometers [21].
B. Primary health centers offer general medicine, primary health care, maternity and childhood services, dentistry, laboratory tests, and X-rays. These centers serve areas with populations exceeding 2,000 residents, and service points should be within a minimum distance of 1 kilometer and a maximum distance of 2 kilometers, as per the Jordanian Ministry of Health's guidelines.
C. Sub-health centers are operational for limited hours and cater to populations in small to medium-sized areas, offering essential medical services exclusively. Sub-health centers are established in areas with populations exceeding 500 people, and the distance between these facilities and residents must not exceed a minimum of 0.5 kilometers or a maximum of 1 kilometer, as prescribed by the Jordanian Ministry of Health.

**Table 1:** Standards for planning comprehensive health centers in Jordan. Source: Ministry of Health

1. Standards for planning comprehensive health centers		
	Minor values	Maximum values
Number of populations served	5000	50000
Distance	2 km (20 min)	5 km
2. Standards for planning primary health centers		
	Minor values	Maximum values
Number of populations served	2000	5000
Distance	1 km (15 min)	2 km
3. Standards for planning Sub health centers		
	Minor values	Maximum values
Number of populations served	500	2000
Distance	0.5 km (7.5 min)	1 km

#### Hospitals

Hospitals offer short-term and long-term medical treatment, encompassing diagnosis, therapy, rehabilitation, and care for ill, injured patients. In Jordan, a diverse array of public and private hospitals is dispersed throughout the nation's governorates, providing vital medical services to the populace.

As of 2020, Jordan boasted a total of 117 hospitals, collectively providing a capacity of 14,378 beds to cater to the healthcare needs of its residents. The distribution of these beds reveals the intricate interplay between various healthcare sectors. Hospitals administered by the Ministry of Health offered 5,251 beds, constituting 35% of the total, while the Royal Medical Services managed 3,154 beds, representing 21% of the country's hospital bed capacity. The private sector contributed substantially to 5,415 beds, comprising 36.1% of the nation's total beds [21].

While this allocation appears substantial, international benchmarks provide an illuminating perspective. According to the World Health Organization (WHO), the global average stipulates a minimum of 28 beds for every 10000 individuals [21]. In 2020, Jordan's healthcare landscape featured 14 beds per 10000 people [33].

This intricate web of health facility types and their respective guidelines forms the bedrock of healthcare accessibility in Jordan, catering to diverse population sizes and healthcare needs while adhering to specific geographical parameters.

## Methods

### Study area

Our research focuses on the Irbid Governorate in Jordan, which was chosen as a case study for several compelling reasons. Irbid is one of the largest cities in Jordan; it is characterized by a high population density and plays a pivotal role as a service center for the northern region of the Kingdom. Demographic shifts across the entire region, stemming from regional conflicts, have significantly influenced Irbid. The influx of migrants into Jordan has notably impacted Irbid, with a substantial return of more than 350,000 individuals to Jordan after the first Gulf War in 1991 [4, 30], and approximately 450,000 Iraqi individuals entering Jordan following the second Gulf War in 2003 [18, 30]. Moreover, a substantial surge in Syrian asylum seekers has augmented the population of Irbid. By 2020, the Irbid Governorate accommodated nearly 2 million residents, positioning Irbid as the second-largest metropolitan population in Jordan, trailing only Amman, thereby highlighting its considerable demographic scale [11]. This surge in population has exerted noteworthy pressure on various services, notably within the healthcare sector.

**Fig. 2:**
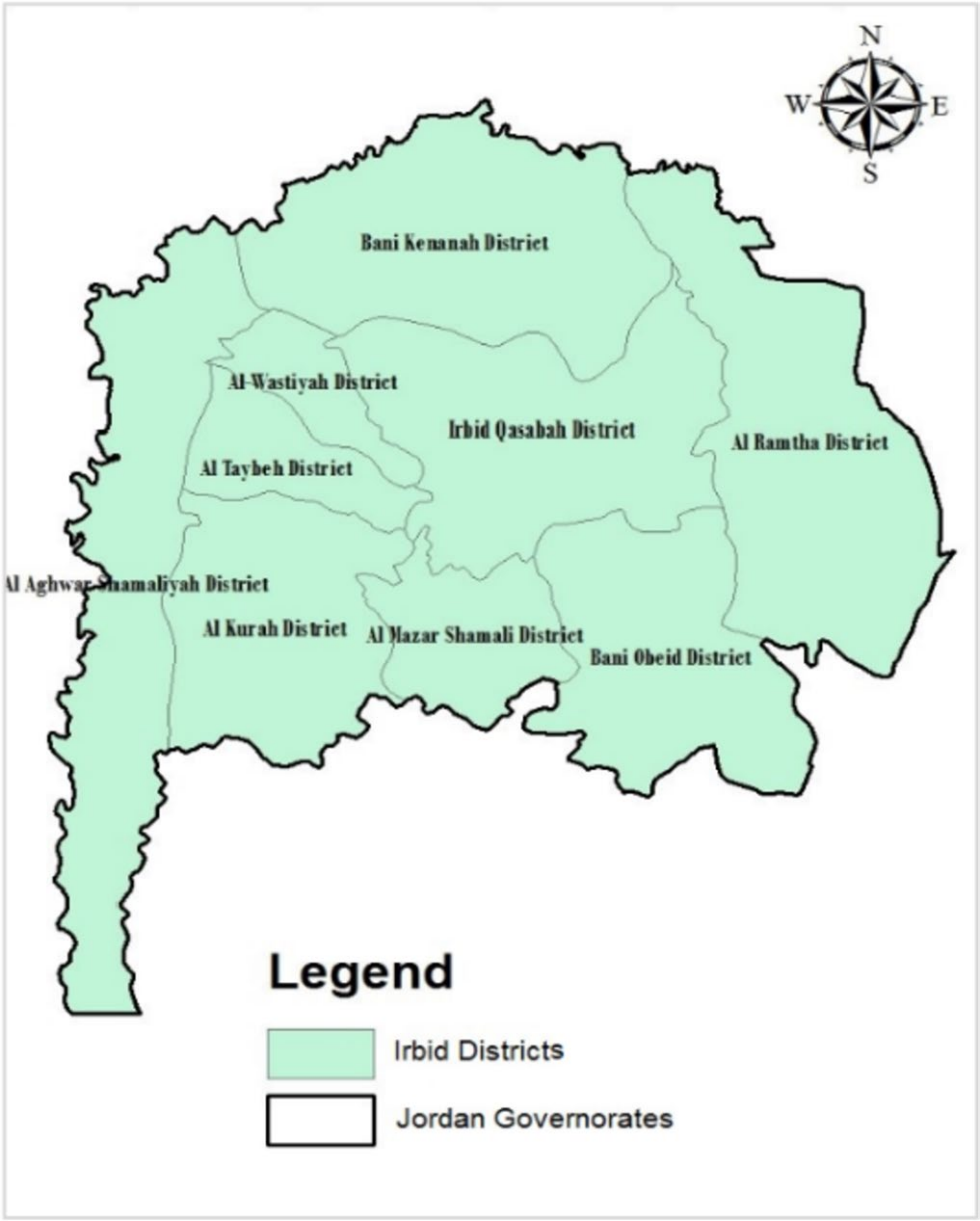
Study area: Irbid Governorate map

Spanning an area of 1,572 square kilometers, the governorate's administrative boundaries are intricately divided into eighteen municipalities and nine districts (Fig. 2). Within the confines of the Irbid Governorate, there is a network of healthcare facilities, including health centers and hospitals under the purview of the Ministry of Health. The total number of public health centers reached an impressive 122, accompanied by 10 public hospitals. The Irbid Governorate provides a unique research opportunity to examine the interplay between population dynamics and healthcare facility distribution.

The urban transformation of Irbid has undergone multifaceted phases over recent decades, resulting in citywide expansion along various axes [3, 4, 6, 30]. These transitions manifest initially within the city's core and subsequently permeate peripheral regions in accordance with the urban hierarchy. Influencing factors include population growth,the evolution of commercial and real estate ventures such as the Al-Hassan Industrial Estate; and the establishment of governmental educational institutions, notably Yarmouk University in 1976 and Jordan University of Science and Technology in 1986 [18]. This confluence has engendered a substantial increase in employment opportunities, consequently driving the demand for infrastructural development and the assimilation of new areas into the city's organizational schema.

**Fig. 3:**
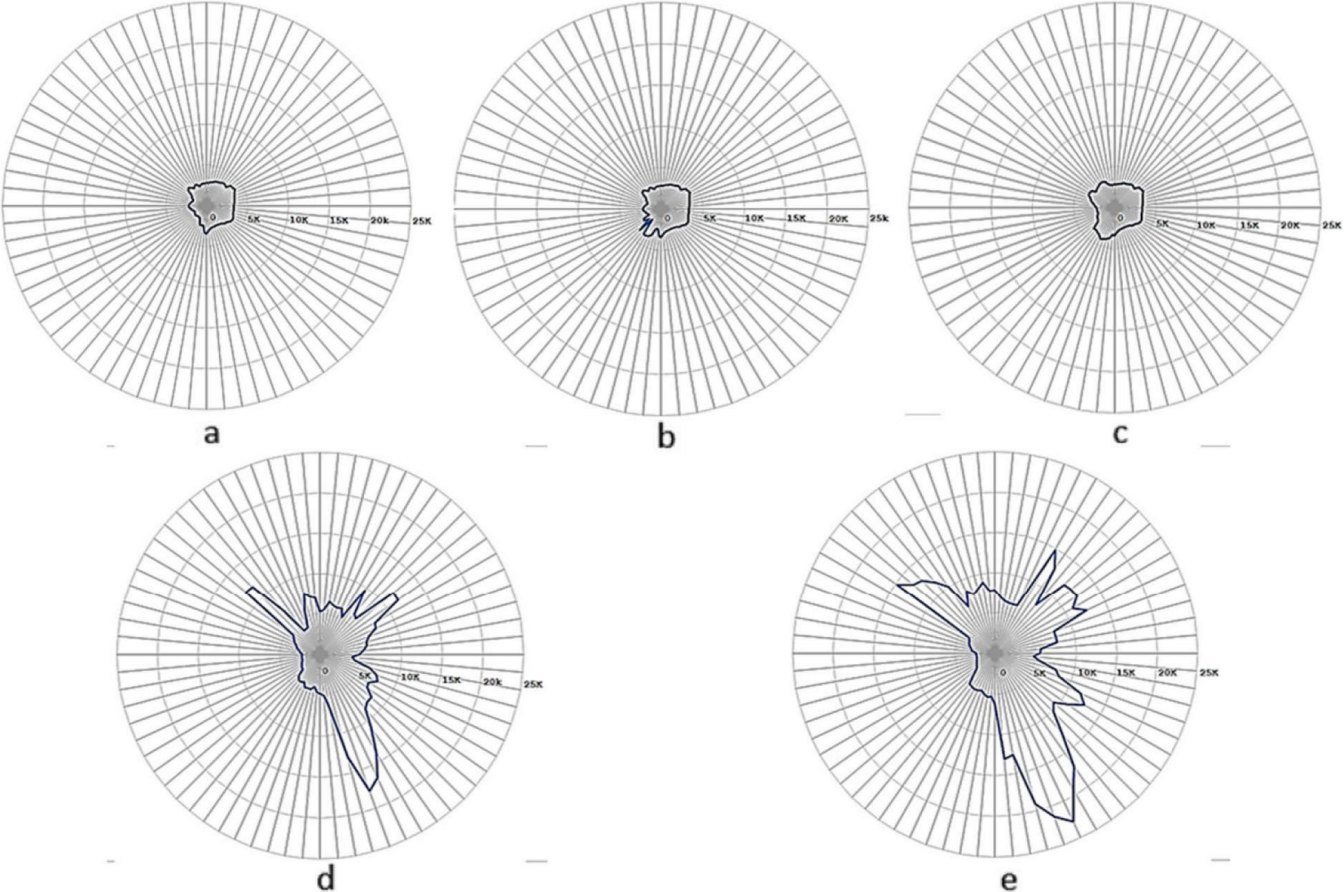
The succession of greater Irbid municipality growth in 1975,1985,1995, 2005, and 2015, (**a**-**e**) respectively. Source [15]

Despite the city's strategic blueprint delineating land use and street network expansion, the effective implementation of these plans has not invariably curtailed urban proliferation and advancement. The phases of Irbid's transformation have been studied by Aldeek and Mistarihi [3], Alwedyan [4], Bagaeen and Hijazi [6], Tarrad [30], and other researchers. According to these studies, the city's developmental chronicle has been observed to unfold across five overarching phases spanning the past half-century as follows (refer to Fig. 3):

- In the 1950s, a systematic expansion occurred in all directions, adopting a radial trajectory emanating from the historical nucleus known as ‘Tal Irbid.’
- In the 1970s, Irbid expanded over an expanse of approximately 18.6 square kilometers, with westward expansion impeded by the natural features of Al-Ghafr valley. Expansion ensued in the northwestern and southern directions, culminating in the emergence of focal points, namely Yarmouk University and the Industrial City neighborhood, enticing population influx.
- In the 1980s, the city’s organized area continued to expand to 25.4 square kilometers. Growth unfolded in three directions - eastward, northwestward, and south-westward, - facilitated by subdivision projects.
- The 1990s marked further expansion, extending the organized area to 30.7 square kilometers. Growth occurred in the north and west directions, coupled with the acquisition of additional zones. A salient characteristic of this phase was the spatial breadth of the industrial city focus.
- By 2010, the city had expanded by 13.9% to cover an area of 35.7 square kilometers. This expansion aimed southward toward Amman, with the objective of establishing the Greater Irbid Municipality (GIM) in 2001. Subsequently, the city maintained its dimensions until 2017, characterized by an administrative nature aligned with the government's decentralization principle.

The city's developmental trajectory has exhibited an irregular pattern marked by unregulated urban sprawl and indeterminate expansion directions during specific stages, posing challenges to effective urban planning [18]. This underscores the imperative for a comprehensive and coordinated approach to urban development and land-use planning.

The rapid evolution of Irbid, which later culminated in the Greater Irbid Municipality, has significantly altered the city's social and urban fabric. The influx of residents, including internal migrants from surrounding villages and individuals of diverse nationalities such as university students, workers, and refugees, has precipitated reactions to the development of road networks and public services. Governmental entities, including ministries and centers, have undertaken studies and provided diverse services within the municipality territory. In contrast, village development has followed a gradual trajectory, with municipal responsibility for service provision contingent on resources and population dynamics. This dynamic diverges from the focus on towns and villages in the Irbid Governorate, highlighting the nuanced nature of healthcare services and developmental priorities.

The urban development of Irbid has intricately shaped the distribution of healthcare services, adapting to the city's expansion and growth over various stages. The accessibility of healthcare has been impacted by city expansion in different directions, necessitating adjustments in the distribution of healthcare facilities to ensure widespread availability. The development of healthcare infrastructure, including hospitals, clinics, and medical centers, has been imperative to meet the escalating needs of the growing population. Urban planning efforts have played a crucial role in strategically locating healthcare facilities to achieve equitable distribution across both the established urban core and newly developed areas, aiming to provide universal access to healthcare services. As the city continues to evolve, this study aims to assess the distribution and coverage of healthcare services, considering the dynamic urban landscape and ongoing demographic changes.

### Data and materials

The study harnessed the capabilities of Geographic Information Systems (GIS) software for the purposes of spatial mapping, graphical visualization, and analytical assessment. Valuable datasets were drawn from official sources, and meticulously selected for relevance and reliability:

- The Department of Statistics, from which comprehensive population and housing census data for both the Irbid Governorate and its individual districts in 2021 were obtained.
- The Irbid Municipality is a key resource that provides essential spatial maps that greatly contribute to the geographical context of the research.
- The Jordanian Ministry of Health offers information on healthcare services and their locations.
- The Ministry of Public Works and Housing provided data concerning the road network within the Irbid Governorate, with the most recent update dating back to 2016.

The dataset included geospatial representations of healthcare facilities represented as point layers, meticulously capturing the precise latitudinal and longitudinal coordinates of these sites. A table of attributes comprehensively cataloged information regarding healthcare services, encompassing details about their classifications, nomenclature, and the nature of the services provided. Furthermore, supplementary layers feature polygons that distinctly delineate the administrative boundaries governing the regions, encompassing districts and governorates.

Additionally, line-based layers meticulously detail the street network, seamlessly weaving through the area of study. Significantly, these layers were augmented with demographic information intricately linked to each respective directorate within the governorate through the utilization of an attribute table.

In this phase, the research advanced to the application of spatial analysis techniques, harnessing the analytical power of GIS. In this study, a combination of spatial analysis techniques was employed to address gaps in knowledge regarding health service distribution in the Irbid Governorate including the calculation of buffer zones, nearest neighbor assessments, and service area demarcations. Nearest neighbor analysis was used to determine the spatial distribution patterns within the dataset, shedding light on clustering tendencies and spatial organization. This method allowed us to assess the degree of randomness or regularity in the distribution of healthcare facilities. Additionally, buffer analysis was employed to delineate health facility coverage distances, considering predefined buffer distances based on Jordanian Ministry of Health standards. This technique facilitated the illustration of the spatial impact and reach of each health facility, accounting for both the minimum and maximum operational ranges. Finally, Service Area Analysis was conducted to evaluate the accessibility of healthcare services within a specified time frame; utilizing a 15-minute access time equivalent to approximately 1000 meters on foot. This method provided a comprehensive understanding of the areas reachable within a specified time or distance frame, considering factors such as road networks and impedance.

The nearest neighbor analysis, buffer analysis, and service area analysis collectively allowed for a systematic assessment of health center distributions and accessibility to the population. This multimethod approach contributes to a nuanced understanding of the spatial dynamics of healthcare services in the Irbid Governorate, addressing key knowledge gaps in the field. The upcoming text offers a comprehensive explanation of each technique employed, supplemented by a comparison presented in Table 2.

**Table 2:** A comparison table of GIS analysis tools employed in this study

**Feature**	**Nearest Neighbor Analysis**	**Buffer Analysis**	**Service Area Analysis**
**Description**	Evaluates spatial distribution by calculating distances between features and their nearest neighbors.	Creates zones or circular areas around geographic features to illustrate their spatial impact or influence.	Determines areas accessible from one or more service points within a network, considering factors like travel time and road networks.
**Purpose**	Analyze the spatial pattern of points (clustered, random, or dispersed)	Create zones of a specified distance around features (points, lines, or polygons)	Define areas accessible within a given time or travel cost from a set of facilities
**Input data**	Point data only	Any type of feature data (points, lines, polygons)	Point data representing facilities, network data for travel paths
**Output data**	Statistical measures of spatial pattern (e.g., nearest neighbor ratio, Ripley's K)	Polygons representing buffer zones around features	Polygons representing areas accessible within the specified time/cost
**Applications**	Identify areas with potential environmental hazards, assess disease clusters, analyze urban development patterns	Site selection for new facilities, determine service areas and operational ranges for existing facilities, based on predefined buffer distances, analyze accessibility to resources	Planning public transportation routes, identifying areas under served by emergency services, analyzing market potential
**Visualization**	Maps with points color-coded by nearest neighbor distances or Ripley's K values	Maps with buffer zones around features	Maps with areas color-coded by travel time/cost from facilities
**Strengths**	Provides insights into patterns within datasets.	Easily visualizes spatial influence of features.	Accounts for real-world travel constraints and provides realistic service coverage.
**Limitations**	Assumes uniform distribution of points in a random pattern, may not be suitable for clustered or dispersed patterns	Sensitive to scale and projection, buffer shapes may not accurately represent real-world accessibility	Relies on accurate network data and travel cost/time models, may not capture individual travel behavior

#### Nearest neighbor analysis

Nearest neighbor analysis, a geospatial methodology, illuminates latent patterns within the spatial distribution of points across a landscape. This analysis provides valuable insights into the spatial organization or randomness inherent in the distribution of objects or events as health facilities within a specific area. This analytical tool quantifies the distances between the centroid of each feature and the centroid of its nearest neighbor, subsequently calculating the average of these nearest neighbor distances. An average distance index below 1 signifies clustering, while an index exceeding 1 suggests a trend toward dispersion [12]. The equations employed to calculate the average nearest neighbor distance index are as follows:

The mean nearest neighbor distance

1 $$\overline{d }=\left(\sum_{i=1}^{N}{d}_{i}\right)/N$$

where N is the number of points ${d}_{i}$ is the nearest neighbor distance for point i.

To conduct a nearest neighbor analysis, the test was chosen from among the spatial analysis tools in GIS. Subsequently, the layer or dataset containing the point features is specified, along with relevant parameters such as the distance calculation method and study area extent. The analysis was subsequently performed to obtain the results, including the nearest neighbor index, z score, and p value. The nearest neighbor index measures the spatial distribution from 0 (clustered pattern) to 1 (randomly dispersed pattern) to 2.15 (regularly dispersed /uniform pattern) [12]. The z score and p value results from the statistical test assess the significance of the findings and guide the decision on whether to reject the null hypothesis. For the average nearest neighbor statistic, the null hypothesis assumes a random distribution of features. It is essential to note that the efficacy of the average nearest neighbor tool relies on the area, where slight adjustments in this parameter can lead to considerable variations in the z score and p value. As a result, the tool is particularly beneficial for comparing diverse features within a uniform study area.

#### Buffer analysis

Buffer analysis is a geospatial technique that involves the creation of zones or circular areas around specific geographic features. These zones, referred to as buffers, serve to illustrate the spatial impact or influence of a geographic feature within a defined radius [13].

In the establishment of buffer zones, the initial step entails the careful selection of pertinent features, which may manifest as points, lines, or polygons, representing diverse entities ranging from educational institutions and medical facilities to roadways and watercourses. Subsequently, the determination of a buffer distance follows, dictating the spatial extent of the created zones. This distance can be uniform or variable and contingent upon specific criteria such as road classifications or land use patterns.

For health facilities, the demarcation of coverage distances involves the application of two distinct buffer zones encircling each facility. These buffers define the operational range, accounting for both the minimum and maximum reaches of a given service. The precise buffer distance is contingent upon the categorization of the health facility, adhering to standards established by the Jordanian Ministry of Health. Notably, comprehensive health centers extend their coverage radius between 2000 and 5000 meters, primary centers operate within a span of 1000 to 2000 meters, and sub-health centers are designed to encompass distances ranging from 500 to 1000 meters. Moreover, hospitals exhibit a coverage area within a radius of 20 km.

Buffer zones are commonly portrayed as polygons on cartographic representations, often employing color-coded or shaded visualizations to underscore their significance. This graphical depiction serves to elucidate the spatial relationships between features and their surroundings, facilitating a comprehensive understanding of their potential impacts. Additionally, the use of buffers aids in the effective visualization of spatial patterns and the assessment of how features interact within their respective environments.

#### Service area analysis

Service area analysis is a spatial analytical technique utilized for visualizing and determining the scope of areas accessible from designated service points within a network [14] and allows for an in-depth examination of accessibility patterns. As an illustration, the service area within a five-minute radius for a specific network point encompasses all the roadways accessible within five minutes from that particular location. Notably, service areas, generated through the Network Analyst, facilitate the assessment of accessibility variations by employing concentric representations that account for impedance factors.

Within the GIS framework, the Network Analyst toolbar affords the capacity to construct a service area analysis layer, incorporating six distinct network analysis classes: facilities, lines, polygons, point barriers, line barriers, and polygon barriers. Following this, the configuration of parameters, such as impedance (e.g., time or distance), cutoff values (maximum limit), and other relevant settings, is essential for tailoring the analysis to specific objectives. Subsequently, the software computes service area polygons, delineating accessible regions from specified facilities within the predefined constraints.

In the context of this study, a 15-minute access time was employed for health facilities, corresponding to approximately 1000 meters on foot. The resulting network service area is represented as a spatial entity encompassing all traversable streets, providing a visual representation of areas reachable within the specified time or distance parameters. This approach aids in comprehending the serviceable area of critical facilities, including hospitals, schools, or businesses, and factors such as travel time, distance, road networks, and impedance variables.

In conclusion, the spatial analyses utilized in this study, encompassing nearest neighbor analysis, buffer analysis, and service area analysis, offer valuable insights into the spatial distribution and accessibility of healthcare facilities. These methodologies contribute to a nuanced understanding of patterns, clusters, operational ranges, and accessibility of healthcare services within the study area, aligning with Jordanian Ministry of Health standards and benchmarks. The following research questions guided this analysis:

- What geographical patterns underlie the distribution of health facilities, including health centers and hospitals, within the Irbid Governorate?
- How well do healthcare facilities conform to Jordanian Ministry of Health standards regarding their operational areas?
- To what extent does the distribution of healthcare facilities in Irbid, meeting the prescribed Ministry of Health planning standards, demonstrate efficiency in terms of their operational areas?
- To what extent does healthcare service distribution, meeting the prescribed Ministry of Health planning standards, ensure equitable access?

### Limitations of the Study

It is important to note that this study has certain limitations that should be considered by the general readership. The maps utilized in this research were obtained from municipal records dating back to 2015, and only three GIS spatial analyses were applied. The data regarding population density and healthcare facilities, however, pertained to the year 2020.

It is important to acknowledge that the findings of this study are context-specific and may not be fully applicable to other areas, given that it is a case study. Further cases can be included to enhance the breadth of the findings.

Additionally, this study focused exclusively on public services, and the scope did not encompass an analysis of private healthcare facilities or other related services.

## Results

**Fig. 4:**
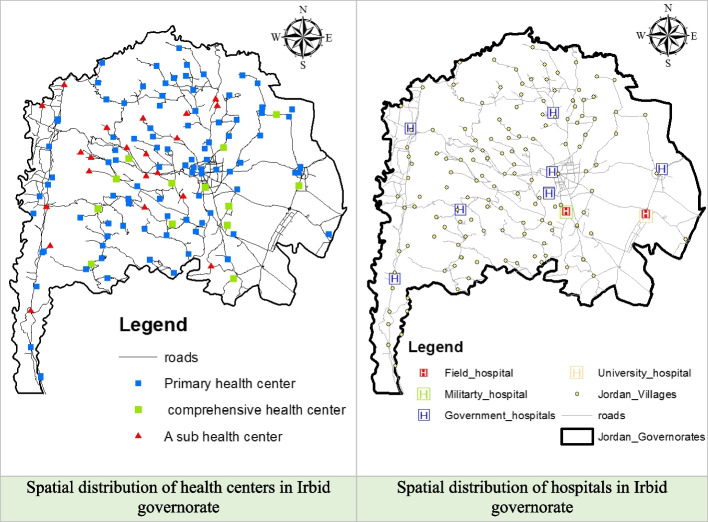
Spatial distribution of health centers and hospitals in the Irbid Governorate

In the Irbid Governorate, the Ministry of Health maintained a consistent number of health centers throughout the entirety of 2020, totaling 122. This figure encompasses 13 comprehensive health centers, 87 primary healthcare centers, and 22 secondary health centers. These health centers are widely distributed and cover all the districts within the Irbid Governorate, as visually depicted in Fig. 4. Notably, a substantial concentration of health centers is observed in the Qasabah Irbid district, functioning as the central hub of the Irbid Governorate. Additionally, a notable concentration is observed in the Bani Kenanah District, which is situated in the northern part of the Governorate.

Within the Irbid Governorate, a total of 17 hospitals, representing the public, private, and charitable sectors, are strategically distributed across the region, as illustrated in Fig. 4.

Table 3 presents data on the number of health centers, district populations, and percentage of health centers per population in various districts of the Irbid Governorate. A comparative analysis with the benchmark established by the Jordanian Ministry of Health, advocating a national average of 10 health centers per 100,000 individuals, revealed discernible variations in healthcare service availability across the different districts in the Irbid Governorate. Some districts, including Bani Kenanah, Aghwar Shamaliyah, Mazar Shamali, Taybeh, and Wastiyyah, surpassed the national benchmark, indicating a heightened density of healthcare centers. Conversely, Qasabah Irbid, Ramtha, Koorah, and Bani Obeid fall significantly below the benchmark, suggesting potential disparities in healthcare availability. The Irbid Governorate as a whole has a healthcare center prevalence of 6.08 per 100,000 residents, significantly trailing both the benchmark and the standards established by the Ministry of Health and the World Health Organization.

**Table 3:** Numbers of health centers based on their spatial distributions in the Irbid Governorate

**Districts of Irbid Governorate**	**Number of health centers**	**Number of Populations**	**Percentage of (Numbers of centers per100000 population)**
Qasabah Irbid	32	836780	3.824183178
Ramtha	12	269980	4.444773687
Koorah	14	182820	7.657805492
Bani Kenanah	21	149190	14.07601046
Aghwar Shamaliyah	14	138480	10.10976314
Bani Obeid	6	231280	2.594258042
Mazar Shamali	10	88780	11.26379815
Taybeh	6	58300	10.2915952
Wastiyyah	7	48190	14.52583524
**Sum**	**122**	**2003800**	**6.088431979**

Source: Ministry of Health, 2020

The Irbid Governorate hosts a total of 17 hospitals, encompassing the public, private, and charitable sectors. Among the 17 hospitals, eight are public hospitals operated by the Ministry of Health, and one of these hospitals specializes in obstetrics and gynecology. In response to the efforts of the Ministry of Health to combat the coronavirus in 2020, two new field hospitals were established to cater to the healthcare needs of patients affected by the virus. One of these new field hospitals is supervised by the Royal Military Services, while the other is affiliated with a university.

According to the guidelines set by the World Health Organization (WHO), a minimum of 29 hospital beds are recommended for every 10,000 individuals [33]. However, according to the Ministry of Health (2020) report, in Jordan, the ratio was 14 beds per 10,000 people in the year 2020. Within the Irbid Governorate, the average ratio of beds to population in public hospitals is 4.3 beds per 10,000 persons. The number rises to 11.3 when accounting for the beds offered by private hospitals, royal medical services, and the university hospital. Nevertheless, neither of these numbers met the criteria established by the WHO or local healthcare authorities, as illustrated in Table 4.

**Table 4:** Distribution of hospitals and the number of beds in Irbid Governorate

**The medical section**	**Number of hospitals**		**Number of beds**		**Percentage hospitals per10000 population**
	**Number**	**The ratio**	**Number**	**The ratio**	
**Ministry of Health**	8	47%	869	38.21%	4.34
**Royal Medical Services**	1	5.88%	448	19.70%	2.24
**University hospitals**	1	5.88%	558	24.53%	2.78
**Private sector**	7	41.17%	399	17.54%	1.99
**Sum**	**17**	**100%**	**2274**	**100%**	**11.35**

Source: Ministry of Health

### Pattern of spatial distribution of public health facilities

**Fig. 5:**
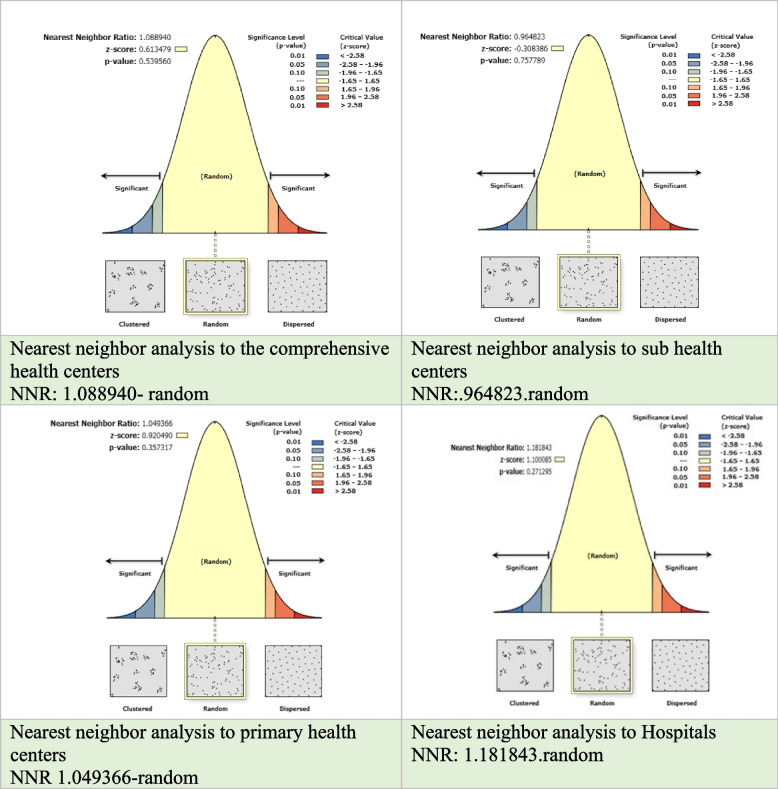
The nearest neighbor ratio (NNR) values for health facilities

The results of the nearest neighborhood ratio (NNR) analysis provide essential insights into the arrangement and dispersion of healthcare facilities, shedding light on the level of order or randomness in their spatial distribution across the Irbid Governorate. The analysis revealed distinct NNR values for different categories of health facilities in the Irbid Governorate, as presented in Fig. 5. The NNR was calculated as follows: 1.088940 for comprehensive centers, 1.049366 for primary centers, 0.964823 for sub-health centers, and 1.181843 for hospitals. These NNR values provide an indication of the spatial distribution of health facilities within the governorate. The findings indicate that the NNR values are approximately 1, suggesting a relatively random distribution pattern of health facilities in the Irbid Governorate. Furthermore, the obtained z scores, which all fall below the threshold of +1.96, and the calculated p values, which collectively exceed 0.05, lead to the conclusion that the null hypothesis of spatial randomness cannot be rejected. These results imply that health facilities in the Irbid Governorate lack a structured spatial pattern and are randomly distributed across the region without any specific factors or decisions determining their location.

### Study of the Services Radius for Health Centers

The results of the buffer analysis have provided valuable insights into the distribution of health centers across the Irbid Governorate (Fig. 5). Comprehensive health center coverage areas are predominantly concentrated in the central and eastern regions, resulting in a significant portion (approximately 37.06%) of the governorate remaining without comprehensive health center coverage. Notably, this deficit disproportionately affects the northern and western areas, notably impacting the villages of Bani Kenanah and Aghwar Shamaliyah. However, these underserved areas still provide essential healthcare services through sub health centers or primary centers.

In contrast, the distribution of primary health centers is more even throughout the governorate, with coverage areas extending to approximately 69.17% of the Irbid region, encompassing most of the districts. Sub health centers, although fewer in number, exhibit the smallest coverage area at 4.19%, which is primarily concentrated in the central and western areas of Irbid. When the coverage areas of comprehensive, primary, and sub-health centers overlap, the combined coverage area reaches 80% of the Irbid governorate. These findings suggested overall good coverage of health centers across the region.

**Fig. 6:**
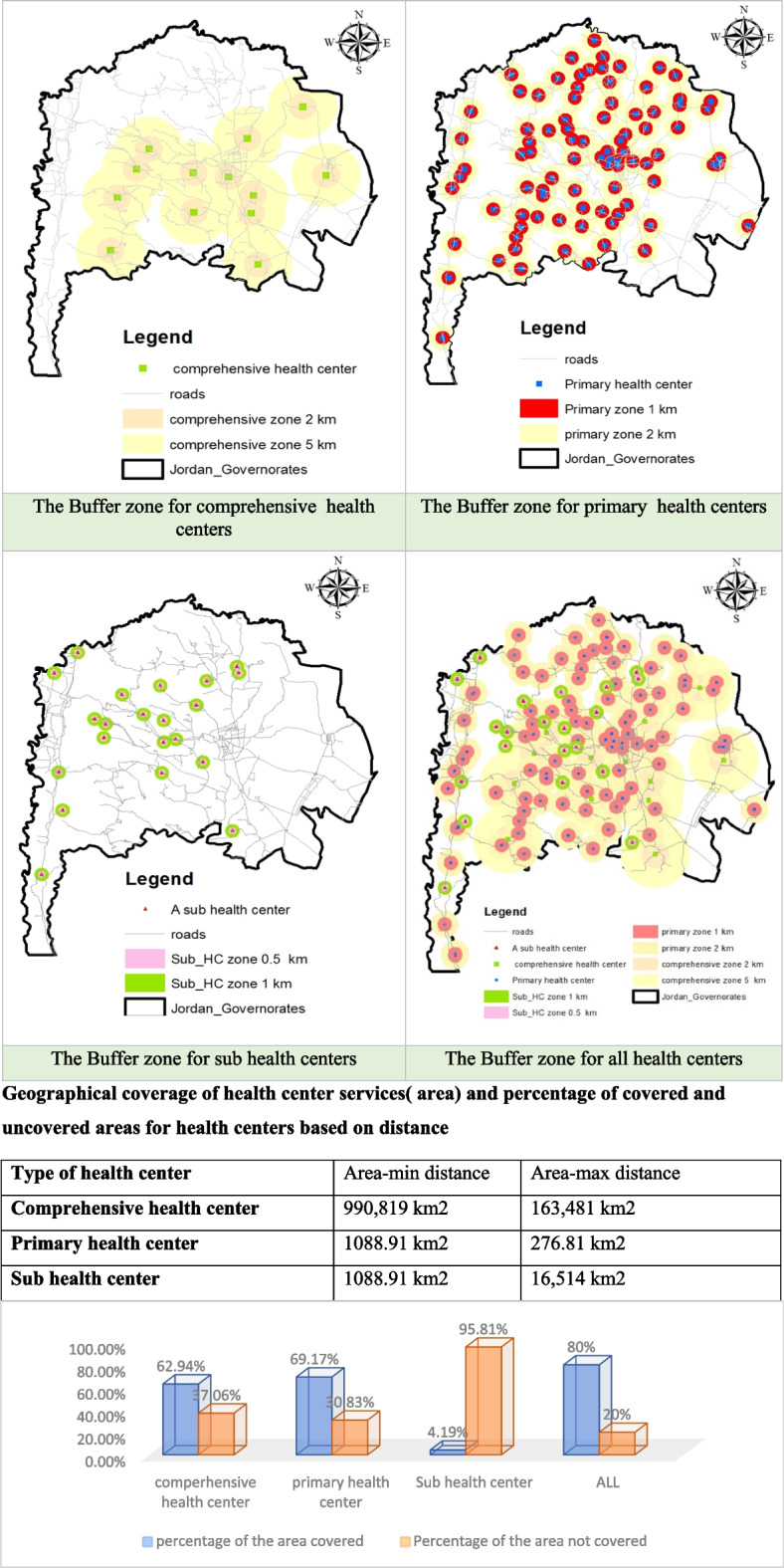
Buffer zones for health centers in the Irbid Governorate and percentages of covered and uncovered areas for health centers based on distance

However, it is essential to highlight that certain villages within Irbid remain underserved, while others exhibit a high density of health centers, particularly in the central part of the governorate. The villages lacking health center coverage include Esheh, Al-Yarmouk, Al-Hemah Aurdinyah, Karima, Abu Ziad, Siaikhat, and Qarn. For a visual representation of these findings, please refer to Fig. 6.

### Buffer analysis for the hospitals

**Fig. 7:**
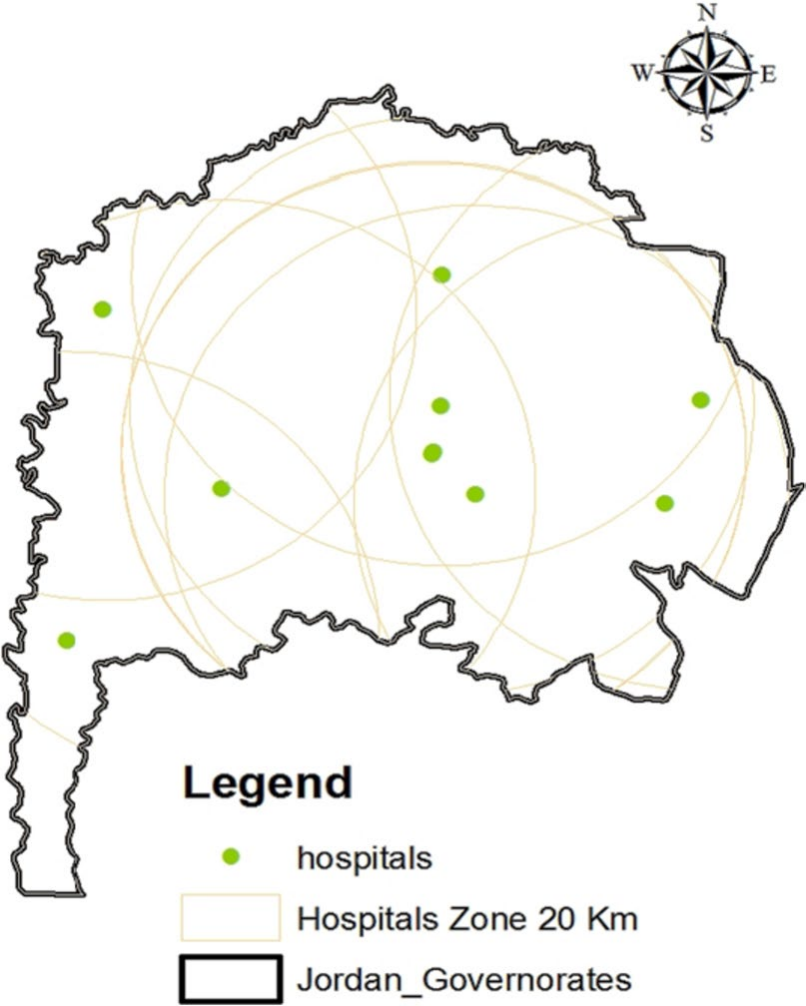
The buffer zone for hospitals in the Irbid Governorate

The Irbid Governorate is served by ten public hospitals, and this study assumes a maximum service radius of 20 kilometers for hospital sites. The results of the buffer analysis, as illustrated in Fig. 7, reveal that hospital care is accessible across a vast expanse of the province. Specifically, hospital services are available in 97.105% of the total area of the governorate, encompassing a substantial 1528,597 square kilometers.

### Evaluation of physical accessibility

**Fig. 8:**
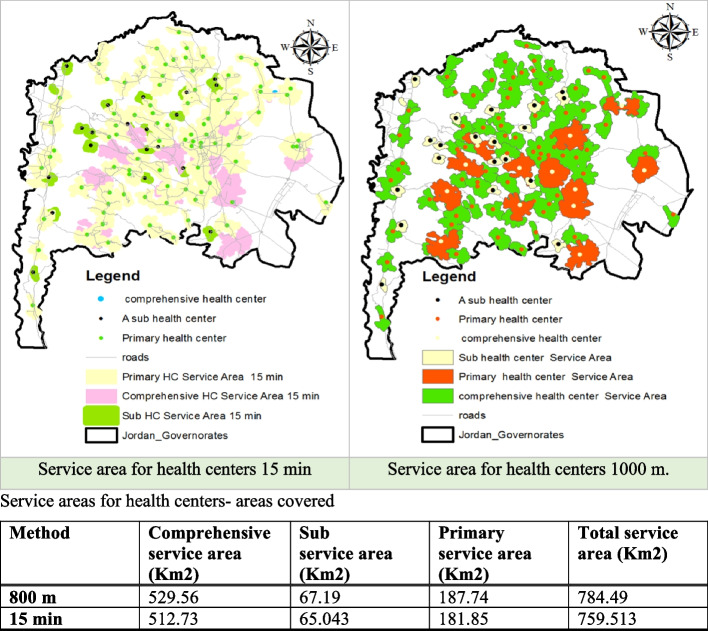
Service areas for health centers

Service area analysis helps determine the physical accessibility of health facilities in the Irbid Governorate; by assessing travel distance and time. The results of the service area analysis suggest that there are areas within the Irbid Governorate where residents may face challenges accessing health centers (Fig. 8). When a 15-minute travel time threshold was used as the measure of accessibility, approximately 51.82% of Irbid's territory was considered inaccessible to health centers, indicating limited access. Similarly, when considering a distance criterion of 1000 meters, almost 50% of the region did not meet the accessibility standard. This implies that residents in these areas may face longer travel distances or spend more time reaching the nearest health centers, potentially impacting their ability to access timely healthcare services.

Some villages experiencing difficulties accessing health centers include Subeirrah, Al Bahwah, Kherbet, Al Hawi, Siras, Al Rahmah, Barasha, Al Yarmouk, Al Esheh, Baqurah, Wadi Al Arab, Irkheim, Sakayin, Al Gurn, Kuraymah, Suleikhat, and Kurkuma. These findings emphasize the need for targeted improvements in healthcare infrastructure and services to ensure equitable access for all residents in these underserved areas.

## Discussion

This study reveals significant insights into healthcare facility distribution and accessibility in the Irbid Governorate, Jordan, utilizing GIS techniques. While the total number of healthcare facilities (122 health centers and 17 hospitals) implies sufficient coverage, comparisons against benchmarks uncover disparities across districts.

A comparison of the number of health centers in the Irbid Governorate with the national benchmark of 10 per 100,000 people, established by the Jordanian Ministry of Health, revealed uneven service distributions across districts. While some districts exceed this benchmark, boasting a higher density of facilities, others fall noticeably short. The concentration of health centers, particularly comprehensive healthcare centers, in the central district (Qasabah district) and Bani Kenanh district indicates a focal point for specialized services in these areas, aligning with their high population density. However, this raises concerns about potential inequities in essential healthcare access, especially in remote regions, despite the presence of sub health centers.

The assessment of hospital bed ratios further underscores the challenges faced by the healthcare infrastructure of the Irbid Governorate. The current ratio of 11 beds per 10,000 residents falls short of both national benchmarks and World Health Organization recommendations. This significant shortfall highlights the need for targeted interventions to enhance healthcare capacity and ensure adequate service provision for the governorate population.

Spatial distribution analysis, particularly nearest neighbor analysis (NNA), suggested a random distribution pattern, highlighting the absence of structured organization in health center allocation. These findings align with the results of other studies, such as Minutha and Jayashree [22], Damashi et al. [10], and Yagoub [37], which observed similar spatial randomness in healthcare facility distributions and interpreted this as potentially being the result of random chance. This unbalanced distribution can be interpreted, especially considering the urban development challenges faced by the city over the past half-century. Irbid development plans often fail to keep pace with population growth, potentially resulting in the haphazard placement of health facilities outside of planned areas.

Buffer analysis reflected the distribution of health centers and their coverage area. The results indicate that health centers, encompassing all types, provide coverage for 80% of the governorate area. However, this metric can be misleading when considering the healthcare services provided by each health center type. An analysis of health center distribution based on healthcare service types reveals the concentration of specialized healthcare centers in specific areas of the governorate, resulting in limited access to such services in other regions. For example, comprehensive health centers are concentrated in the central and eastern regions, leading to underserved areas in other parts of the governorate. The skewed distribution of specialized or higher-quality healthcare centers within the Irbid Governorate highlights the need to redistribute facilities more fairly based on their type. This approach will help ensure that essential healthcare needs are adequately addressed in all districts. Furthermore, the decreasing concentration of facilities as one moves away from the central part of the governorate results in lower coverage of public health facilities for residents residing farther from the central areas. This aligns with challenges identified in studies conducted in other cities in Jordan [2, 26] and in developing countries [9, 19, 23].

Accessibility, another crucial metric, provides a nuanced picture. Service area analyses revealed significant shortcomings, with almost half of the Irbid areas falling outside the 15-minute travel time threshold for healthcare centers, particularly in remote regions. Approximately 50% of the governorate area lacks coverage within a 1000-meter radius. These findings resonate with the concerns raised in Maslamani et al. [20], which emphasizes the impact of accessibility on timely healthcare services in Irbid. The challenges of accessibility align with studies on Irbid's urban development, which highlight irregular and fragmented street networks as major barriers [6]. This network, often resulting from organic or unplanned development, significantly extends travel times, potentially exacerbating disparities in access to essential healthcare services, particularly for vulnerable populations [5].

The disparities identified in this study are not exclusive to Irbid alone but resonate with issues faced by many Jordanian governorates, including Mafraq [29], Karak [26], and Jerash [25]. The study by Qutaishat in Jerash, for instance, confirms the presence of villages outside the reach of health centers, underlining nationwide accessibility concerns. In Mafraq, Taran and Alfanatsah's study revealed similar challenges, emphasizing the need for nationwide attention to healthcare distribution. Irbid's relative advantage, with its ten hospitals, helps mitigate the imbalance in health center distribution. However, the issue remains pervasive across Jordan.

Our findings also resonate with a growing body of research documenting similar patterns of healthcare disparity, particularly in developing countries [2, 9, 10, 19, 23]. Mansour [19] highlighted the stark contrast between urban centers teeming with healthcare facilities and rural areas left underserved. Damashi et al. [10] emphasize how resource limitations and transportation inadequacies impede access for disadvantaged populations, while Al-Habees [2] reveal the uneven distribution of specialized services, leaving rural communities particularly vulnerable. This broader tapestry emphasizes the urgent need for context-specific interventions to address these persistent inequities.

The unequal distribution of healthcare facilities in Irbid can be attributed to a complex interplay of various factors. Over the past half-century, urban development challenges have led to rapid population growth, outpacing the implementation of effective development plans. This has resulted in haphazard placement of health facilities, often outside of planned areas, contributing to disparities in accessibility. Economic disparities across districts may lead to variations in healthcare resource density and service quality, with more economically developed urban centers contrasting with less affluent rural areas. Additionally, government policies and regulations, including decisions regarding funding, standards, and incentives, can result in variations in healthcare access across districts. The diverse geography of Irbid, characterized by mountains, valleys, and rugged terrain, poses challenges for healthcare accessibility, with natural barriers limiting the establishment of facilities in specific areas. The existing infrastructure, including transportation networks, also plays a vital role. Areas with well-developed infrastructure are better connected, while less accessible regions experience limited access. Recognizing and addressing these multifaceted factors is crucial for devising effective strategies to improve healthcare distribution and ensure equitable access to healthcare services for all residents.

This research has significant implications for healthcare planning and policy in Irbid, Jordan. The spatial analysis conducted in this study successfully identified disparities in healthcare facility distribution and accessibility, highlighting areas for targeted improvement to ensure equitable access to essential services. Adjusting resource allocation based on population density and health needs becomes imperative for achieving a more balanced distribution of services. Additionally, the proactive integration of healthcare considerations into future urban development plans can optimize facility placement and enhance accessibility. Engaging affected communities in planning and decision-making processes is essential for empowering them to advocate for their specific needs, ensuring that interventions are not only effective but also culturally relevant. Furthermore, the findings contribute valuable insights to the broader understanding of healthcare disparities in similar regions, offering knowledge that can inform context-specific interventions to address persistent challenges in healthcare distribution and access.

## Conclusions

In conclusion, this research has shed light on the distribution and accessibility of healthcare facilities within the Irbid Governorate, Jordan. A systematic examination of health centers in relation to population numbers revealed variations across different districts, indicating the need for tailored resource allocation. Nearest neighbor analysis revealed a random spatial distribution pattern, indicating a lack of structured arrangement in health center locations.

Furthermore, buffer analysis highlighted the concentration of comprehensive health centers in the central and eastern regions, leaving some regions underserved. However, when considering the overlapping coverage of comprehensive, primary, and sub health centers, approximately 80% of Irbid Governorate's area exhibited good overall health center accessibility.

Service area analysis, which focused on travel time and distance, revealed potential accessibility challenges, with a significant portion of the districts within the governorate falling outside the 15-minute threshold. This highlights the necessity of enhancing healthcare infrastructure and services in specific villages and regions.

When benchmarked against international standards, the study revealed that the health resource distribution and accessibility of the Irbid Governorate did not meet the criteria set by the World Health Organization or the Jordanian Ministry of Health. With a rate of 6.08 health centers per 100,000 residents and a hospital bed ratio of 4.3 per 10,000 citizens (for public hospitals), there is room for improvement to ensure equitable access to healthcare.

In light of these findings, it is evident that healthcare resource distribution in the Irbid Governorate requires a more structured and needs-based approach to address disparities in accessibility. This research serves as a valuable resource for healthcare planners, policymakers, and stakeholders to formulate strategies that enhance healthcare access, distribution, and quality, ultimately leading to improved well-being and health outcomes for the residents of the Irbid Governorate. Further studies and interventions are warranted to address these challenges and promote healthcare equity in the region.

## Data Availability

All the data or models that support the findings of this study are available from the corresponding author upon reasonable request.
